# Investigating the association between family connectedness and self-control in adolescence in a genetically sensitive design

**DOI:** 10.1007/s00787-020-01485-9

**Published:** 2020-02-05

**Authors:** Yayouk E. Willems, Odilia M. Laceulle, Meike Bartels, Catrin Finkenauer

**Affiliations:** 1 Department of Biological Psychology, Vrije Universiteit Amsterdam, Boechorststraat 7-9, 1081 BT Amsterdam, The Netherlands; 2 Amsterdam Public Health Research Institute, Vrije Universiteit Amsterdam, Amsterdam, The Netherlands; 3 Department of Interdisciplinary Social Science, Universiteit Utrecht, Utrecht, The Netherlands; 4 Department of Developmental Psychology, Universiteit Utrecht, Utrecht, The Netherlands; 5 Neuroscience Campus Amsterdam, Amsterdam, The Netherlands

**Keywords:** Family connectedness, Self-control, Adolescence, Twins, Genetics, Environment

## Abstract

Family connectedness is key for the development of self-control in early and middle childhood. But is family connectedness still important during the transitional phase of adolescence, when adolescents demand more independence from their parents and rely more on their peers? The aim of the present study was to investigate the association between family connectedness and self-control, and whether it still holds in adolescence using a genetically sensitive design. Data were used from a large sample of twins aged 14 (*N* = 11,260) and aged 16 (*N* = 8175), all enrolled in the Netherlands Twin Register. We applied bivariate twin models and monozygotic twin difference models to investigate the association between family connectedness and self-control and to unravel to what extent genetic and environmental factors explain this association. The results showed that more family connectedness is significantly related to better self-control in adolescence, albeit with a small effect size. Twin analyses revealed that this association was mainly explained by common genetic factors and that the effects of environmental factors were small. The current findings confirm the role of family connectedness in adolescent self-control. Importantly, however, the results demonstrate that phenomena we see within families seem the product of parent and children sharing the same genes rather than being exclusively attributable to environmental processes.

**Electronic supplementary material:**

The online version of this article (10.1007/s00787-020-01485-9) contains supplementary material, which is available to authorized users.

## Introduction

Family connectedness encompasses the feeling of trust, understanding, and support within the family, and is robustly associated with healthy child development [13, 25]. In other words, family connectedness is the emotional connection within the family, also referred to as family warmth or responsiveness, and capturing the way family members give emotional support, show affection and strengthen family bonds [13]. Children develop over time, and so does the influence of the family on the development of the child [57]. This development is especially pronounced during adolescence, as adolescents increasingly become active agents in their own development, demand more independence from their parents, and rely more on connectedness with peers than on connectedness with the family [10]. The developmental transition from being dependent on parents to independence yields an important question: is family connectedness still related to person characteristics in adolescence and, if so, what is the nature of this association? Thus far, most of the researches examining this question focused on early and middle childhood rather than adolescence. Moreover, few studies take the possibility of genetic confounding into account. Unraveling the underlying genetic and environmental mechanisms is important to understand how adolescents develop within, and in interaction with, their social world [30, 49].

### Self-control

A key person characteristic in adolescent development is self-control. Self-control is the capacity to alter unwanted impulses and behaviors to bring them into agreement with internal and external standards [19, 23]. Self-control is proposed to be especially important during adolescence, as adolescents with high self-control capacities have higher-quality interpersonal relationships, better school grades, healthier lifestyles, less psychological problems, and report more happiness than their adolescent peers with less self-control capacities [11, 22, 41]. Given these findings, self-control has also been coined a hallmark for adolescents to become well-adjusted adults [10, 11, 41]. As such, it is important to understand causes and consequences of individual differences in adolescent self-control capacities.

### The association between family connectedness and self-control

Research in early and middle childhood shows that family connectedness and self-control are associated (with associations around *r* = 0.20, see [46]). When parents create a context where family members feel accepted and supported, children get the opportunity to learn how to self-regulate their behaviors and impulses [7, 46]. Additionally, family connectedness is closely related to higher-quality parenting which, in turn, affects children’s opportunities to learn how to self-regulate their impulses, behaviors, and emotions [58]. Importantly, the association between family connectedness and self-control can also be explained by a child’s level of self-control evoking certain family responses. For example, children with high self-control elicit trust, warmth, and affection from their parents and siblings [9], feelings which strengthen family connectedness [59]. Thus, there seems to be a reciprocal association between family connectedness and a child’s self-control.

Research in adolescence on the association between family connectedness and self-control is more inconclusive. For example, while some longitudinal studies in adolescents find no significant bidirectional association [15, 43], others find significant associations with small effect sizes (*r* = 0.15–0.20, [34, 59]), and again others find moderate effect sizes for the association between family connectedness and self-control (*r* = 0.30–0.35, [31]). Important to note is that, even small changes in self-control levels can affect the developmental trajectory of individuals [11].

A particular problem in the current literature is that the associations reported in the studies are commonly interpreted as reflecting causal effects between family connectedness and self-control. Most of these studies, however, are correlational and thereby do not necessarily provide evidence of true direction of effect. An alternative explanation for the association between family connectedness and self-control is that underlying factors influence them both (i.e., no direct causal relationship but a third underlying factor that drives the association between the two, [4, 27]). One key underlying factor may be genetic factors.

### Genetic influences on family connectedness and self-control

Over the last decade, accumulating research shows that traits are at least partly heritable [51]. For example, a recent meta-analysis showed that the heritability of self-control is 60%, with stable heritability estimates across middle childhood and adolescence [64]. Traits closely related to self-control show similar heritability estimates such as grit (37%, [55]), effortful control (49%, [67]), delay discounting (51%, [1]), and attention skills (70%, [52]). Importantly, not only person characteristics, but also contextual factors are partly influenced by genetic factors. Genes do not in any direct way “code” people for specific environments, however, individual’s genetic make-up influences their perception and selection of contexts [49].

The heritability of family connectedness ranges between 30 and 40% [61], and the way children perceive parenting is correlated with their genes (gene–environment correlation) [17, 26, 28].

Considering the genetic contribution to both family connectedness and self-control, it may thus well be that their observed associations are explained by common genetic factors that simultaneously influence both family connectedness and self-control [2, 48, 56]. Thus far, however, studies on the association between family connectedness and self-control specifically applying genetically sensitive designs are scarce. Two studies using a genetic sensitive design found no significant association between parental socialization and self-control in adolescence after controlling for genetic influences. Yet, the study had limited statistical power to solidify these assumptions (monozygotic twin pairs *N* = 289, dizygotic twin pairs *N* = 452, [4, 66]). To further our knowledge, it is important to assess whether family connectedness and self-control are the result of a true directional effect or, alternatively, by a confounding third factor such as common genetic factors simultaneously influencing both the family context and child outcomes (genetic pleiotropy, [48]).

One design allowing researchers to investigate the association between family connectedness and self-control in adolescents, while simultaneously unraveling to what extent the association is influenced by genetic or environmental factors, is the bivariate twin design. This design is built upon the premise that monozygotic twin pair correlations (100% genetically identical) and dizygotic twin pair correlations (on average 50% genetically identical) can be parsed into environmental and genetic influences [6].

Additionally, twin data allow researchers to apply a monozygotic difference design [3, 12, 16]. If there is a causal relationship between family connectedness and self-control, it could be expected that in genetically identical twins, the twin who reported more family connectedness has higher self-control than his/her co-twin, or vice versa. Applying both twin designs, the bivariate twin design and the monozygotic difference design, thereby allows us to better understand the nature of the association between family connectedness and self-control.

### Current study

Previous studies have mainly focused on the association between family connectedness and self-control in middle and early childhood. We aim to extend this line of work to adolescence, a transformative phase for families, parents, and children. Additionally, few studies thus far have investigated the association between family connectedness and self-control in a genetically sensitive design. Such a design can provide information on the extent to which environmental or genetic factors influence this association. The goal of this study is therefore to investigate the nature of the association between family connectedness and self-control. We aim to do so by investigating the following three sub-questions in a large longitudinal sample of adolescent twins aged 14 years and aged 16 years: (1) are family connectedness and self-control associated over the course of adolescence? (2) to what extent is the association explained by genetic or environmental influences? (3) can we determine the directionality of the association between family connectedness and self-control?

## Method

### Sample and procedure

Longitudinal survey data were collected in twins registered with the Netherlands Twin Register, a population-based study initiated in 1987 in the Netherlands at the Vrije Universiteit Amsterdam. Upon parental consent, 14- and 16-year-old twins received questionnaires on family functioning, physical health, and psychological well-being (see [60, 61] for more details on data collection). Data collection was approved by the Medical Ethical Committee at the Vrije Universiteit Medical Center (2003/182).

The dataset comprised 14-year-old twins (57.6% females, MZ twin pairs *N* = 1,905, DZ twin pairs *N* = 3,353), and 16-year-old twins (58.1% females, MZ twin pairs *N* = 1,483, DZ twin pairs *N* = 2,476). See Table S1 Supplemental Material for an overview of the sample size for each effect size. For 28.1% of the same-sex twin pairs, zygosity was determined based on DNA typing or blood group. For the remaining same-sex twins, zygosity was determined based on questionnaire items filled in by parents (e.g., “is it difficult to discern the two siblings from one another”), resulting in accurate determination of zygosity in 93% of the cases [54].

### Measures

*Family connectedness* was assessed with an adolescent self-report subscale of the McMaster Family Assessment Device (FAD) [21]. We used the Dutch translation which shows good psychometrical properties [61, 62], a one-factor structure, and a Cronbach’s alpha coefficient of 0.84 at age 14 and 0.85 at age 16. The subscale consisted of six items tapping into family connectedness such as, “in times of crisis, we can turn to each other for support”, “we feel free to express our feelings within the family”, and “we trust each other”. Items were scored on a 4-point scale, ranging from 1 = strongly disagree to 4 = strongly agree. Scores on individual items were summed, so that higher scores reflected more family connectedness.

*Self-control* was assessed with the adolescent self-report of the ASEBA Self-Control Scale (ASCS) [63]. This scale is shown to be psychometrically sound to assess self-control [63], with a one-factor structure, good test–retest reliability (test–retest correlations of 0.55), and a Cronbach’s alpha coefficient of 0.73 at age 14 and 0.70 at age 16. The scale consists of eight items tapping into self-control with items such as “I fail to finish things that I start” and “I am inattentive or easily distracted”. The response format of the items is a 3-point scale, with response options not true (coded 0), somewhat or sometimes true (coded 1), and very true or often true (coded 2). We recoded the items such that higher sum scores reflected higher overall levels of self-control.

## Statistical analyses

All analyses were conducted in M*plus* version 7 [45], evaluating goodness of fit using the root mean square error of approximation (RMSEA) and the comparative fit index (CFI) with the cutoff scores defined by Hu and Bentler [33]. We compared the fit statistics of different models. If the constrained model (i.e., the more parsimonious model) showed better fit statistics (lower RMSEA, higher CFI) or a non-significant *p* value to the *χ*^2^ test (meaning that the fit of the constrained model was not significantly worse than the fit of the more complex model), we considered this model to better represent the data. Considering our multiple tests (phenotypic correlations, cross-sectional twin analyses at age 14, cross-sectional twin analyses at age 16, longitudinal twin analyses self-control at age 14 to family connectedness at age 16, and longitudinal twin analyses family connectedness at age 14 and self-control at age 16), we applied the more conservative alpha level of 0.01. We applied structural equation modeling (SEM) for all our analyses, applying maximum likelihood estimator (MLR) and controlling for non-independence of observations by clustering data around the family identification variable [53].

### Bivariate twin analyses

**Fig. 1:**
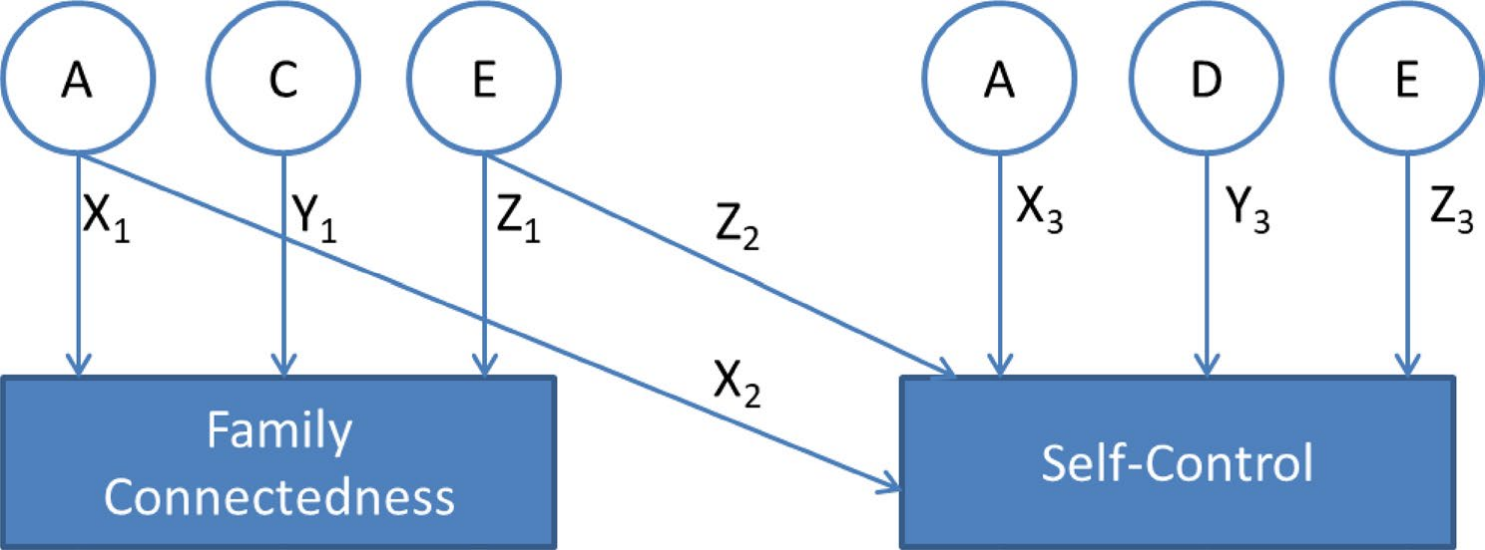
Graphical representation of the longitudinal bivariate twin models. Bivariate Cholesky model with A (additive genetic), C (shared environmental), and E (non-shared environmental) latent variables predicting family connectedness (via path X_1,_ Y_1_, Z_1_), and A, D (dominant genetic) and E explaining self-control (via path X_3,_ Y_3_, Z_3_). We allowed the cross-trait covariance to be through AE (via path X_2,_ Z_2_). Additionally, it can be used to elucidate to what extent family connectedness and self-control are explained by the same genetic factors (the genetic correlation, Rg) and environmental factors (the environmental correlation, Re)

We applied bivariate twin analyses to investigate to what extent genetic and environmental factors contribute to family connectedness and self-control in adolescence. The observed phenotype and the association between two phenotypes can be decomposed into (1) genetic effects which can be additive genetic effects (A), and/or dominance genetic effects (D), (2) shared environmental effects (C), and (3) unique environmental effects (E), which is the part of the total variance that is unique to a certain individual, and also includes measurement error (see Fig. 1). Additionally, it allows us to calculate genetic (*r*_g_) and environmental correlations (*r*_e_), which quantify the extent to which an association is influenced by the same genes or by the same environmental factors (see Fig. 1).

### Monozygotic twin difference model

To further explore direction of causation between family connectedness and self-control in adolescence, we applied monozygotic within-individual change model. This is a method to test whether the monozygotic twin who reports an increase in family connectedness from age 14 to 16 shows an increase in self-control from age 14 to 16, as compared to the co-twin with a lesser increase in family connectedness over time. Because monozygotic twins are genetically identical, any phenotypic difference between them cannot be the result of genetic influences. This model is therefore particularly strong, because it examines an association controlling for genetic confounding and under the causal hypotheses these monozygotic differences should be significantly associated [3, 16].

## Results

### Descriptives

Means, standard deviations, and sample sizes are provided in Table 1. There were no significant mean or variance differences between boys and girls, nor between monozygotic and dizygotic twins. Constraining the twin correlations to be equal across gender did not deteriorate model fit, indicating the absence of gender differences in the genetic architecture of family connectedness or self-control, respectively (see Table S2, Supplemental Material). Most twin pairs consisted of complete pairs (age 14 82% complete MZ twin pairs, age 16 74% complete DZ twin pairs, 77% complete MZ twin pairs, 60% complete DZ twin pairs). Of the adolescents participated at age 14, 54% participated at age 16.

**Table 1:** Means, standard deviations, and phenotypic correlations with 95% confidence intervals

#	Variable	Age	*M*	SD	*N*	1	2	3
1	Family connectedness	14	19.42	2.78	10,685			
2	Self-control	14	11.73	2.76	11,260	0.21* [0.19, 0.23]		
3	Family connectedness	16	19.04	2.87	7430	0.42* [0.38, 0.45]	0.15* [0.12, 0.18]	
4	Self-control	16	11.64	2.69	8175	0.17* [0.14, 0.20]	0.58* [0.56, 0.60]	0.19* [0.17, 0.21]

^*^p < 0.01

### Is there an association between family connectedness and self-control in adolescence?

The phenotypic correlations between family connectedness and self-control are presented in Table 1. All correlations were small, positive, and significant, with a cross-sectional correlation of *r* = 0.21, *p* < 0.001, 95% CI [0.19, 0.23] at age 14, and a cross-sectional correlation of *r* = 0.19, *p* < 0.001, 95% CI [0.17, 0.21] at age 16. The longitudinal association between family connectedness at age 14 and self-control at age 16 was *r* = 0.17, *p* < 0.001, 95% CI [0.14, 0.20]. The longitudinal association between self-control at age 14 and family connectedness at age 16 was *r* = 0.15, *p* < 0.001, 95% CI [0.12, 0.18].

These results suggest that family connectedness and self-control are associated in adolescence, with more family connectedness associated with higher adolescent self-control and, vice versa, higher adolescent self-control associated with more family connectedness. Thus, on average, in families where adolescents report more family connectedness, adolescents report higher self-control and, vice versa, adolescents with higher self-control report their families to be more connected, albeit with a small effect size.

### What are the genetic and environmental contributions to this association?

*Twin correlations.* The twin correlations are summarized in Table 2. All correlations were significant, and all the monozygotic twin correlations were stronger than the dizygotic twin correlations, suggesting the presence of genetic influences on both family connectedness and self-control. For family connectedness, the dizygotic twin correlations were close to the monozygotic twin correlations, which suggests an influence of the shared environment (C), while for self-control the monozygotic correlations were more than twice as high as the dizygotic correlations, indicating the presence of genetic dominance (D).

**Table 2:** Twin correlations and cross-twin cross-trait correlations with 95% confidence intervals

Correlations	MZ	DZ
Twin correlations		
Family connectedness at age 14	0.35*[0.31, 0.39]	0.25*[0.21, 0.29]
Self-control at age 14	0.49*[0.46, 0.53]	0.18*[0.14, 0.22]
Family connectedness at age 16	0.39*[0.34, 0.44]	0.24*[0.19, 0.29]
Self-control at age 16	0.47*[0.43, 0.51]	0.18*[0.13, 0.23]
Cross-twin cross-trait correlations		
Family connectedness at age 14–self-control at age 14	0.18*[0.15, 0.20]	0.09*[0.07, 0.10]
Self-control at age 16–family connectedness at age 16	0.12*[0.09, 0.16]	0.06*[0.04, 0.08]
Family connectedness at age 14–self-control at age 16	0.17*[0.14, 0.21]	0.09*[0.06, 0.11]
Self-control at age 14–family connectedness at age 16	0.11*[0.06, 0.15]	0.05*[0.03, 0.08]

^*^p < 0.01

*Bivariate twin model.* The results of the bivariate genetic twin model are presented in Table 4 (univariate standardized A, C/D, E estimates for family connectedness and self-control, respectively, are presented in Table 3). Full twin models were used in the bivariate twin analyses to avoid biases due to the drop of parameters (that is, we kept ACE for family connectedness and ADE for self-control). For the bivariate estimations, it is not possible to estimate both C and D in the same model, so in line with the classical twin model we estimated an AE model for the overlap between family connectedness and self-control. We modeled the bivariate genetic, and unique environmental effects but not the bivariate shared environmental effects, because it is not possible to estimate both C and D in the same model, and shared environmental influences did not contribute to variation in self-control [65]. All bivariate twin models showed good model fit (see Table S3, Supplemental Material).

**Table 3:** Additive genetic (*A*), dominant genetic (*D*), common shared environmental (*C*), and unique environmental (*E*) estimates [95% confidence interval] to family connectedness and self-control, respectively

Age	Family connectedness			Self-control		
	*A*	*C*	*E*	*A*	*D*	*E*
Age 14	0.19 [0.08, 0.31]	0.15 [0.07, 0.24]	0.65 [0.61, 0.70]	0.22 [0.08, 0.37]	0.27 [0.11, 0.43]	0.51 [0.47, 0.54]
Age 16	0.30 [0.16, 0.45]	0.09 [− 0.02, 0.20]	0.61 [0.56, 0.66]	0.23 [0.04, 0.43]	0.23 [0.03, 0.44]	0.53 [0.49, 0.58]

For the cross-sectional associations, genetic factors largely contributed to the association between family connectedness and self-control. At age 14, the bivariate heritability between family connectedness and self-control was 83%, *p* < 0.001, 95% CI [0.71, 0.94], with the remaining 18%, *p* < 0.01, 95% CI [0.06, 29] explained by unique environmental factors. At age 16, the bivariate heritability was 66%, *p* < 0.001, 95% CI [0.50, 0.83] with the remaining 34%, *p* < 0.001, 95% CI [0.17, 0.50] explained by unique environmental factors.

For the associations from age 14 to 16, genetic factors solely contributed to the link between family connectedness and self-control. The bivariate heritability between family connectedness at age 14 and self-control at age 16 was 95%, *p* < 0.001, 95% CI [0.76, 0.100]. Environmental effects on the covariance were non-significant: 5%, *p* = 0.96, 95% CI [− 0.23, 0.24]. Similarly, the bivariate heritability between self-control at age 14 and family connectedness at age 16 was 72%, *p* < 0.001, 95% CI [0.47, 0.97]. Environmental effects on the covariance were non-significant: 28%, *p* = 0.03, 95% CI [0.03, 0.54].

While the bivariate heritability estimates elucidate the contribution of genetic and environmental factors to the phenotypic association between family connectedness and self-control, the genetic correlations (*r*_g_) and environmental correlations (*r*_e_) quantify the extent to which the two are influenced by the same genes or by the same environmental factors. These correlations are presented in Table 4 and showed that *r*_g_ ranged between 0.36 and 0.63 while *r*_e_ ranged between 0.00 and 0.07. This indicates that, over and above the strong influence of genes on the association between family connectedness and self-control, there is also overlap between the genes involved in both traits. The overlap between environmental factors is small or close to zero.

**Table 4:** Genetic and environmental contributions (95% confidence interval) to the association between family connectedness and self-control

	*A*	*E*	*r*_g_	*r*_e_
Cross-sectional				
Family connectedness at age 14–self-control at age 14	0.83*[0.71, 0.94]	0.18*[0.06, 0.29]	0.63*[.40, 0.86]	0.06*[0.02, 0.10]
Family connectedness at age 16–self-control at age 16	0.66*[0.50, 0.83]	0.34*[0.17, 0.50]	0.37*[0.21, 0.52]	0.10*[0.05, 0.15]
Longitudinal				
Family connectedness at age 14–self-control at age 16	0.95*[0.76, 1.00]	0.05 [− 0.23, 0.24]	0.57*[0.34, 0.81]	0.00 [− 0.07, 0.07]
Self-control at age 14–family connectedness at age 16	0.72*[0.47, 0.97]	0.28 [0.03, 0.54]	0.36*[0.20, 0.57]	0.07 [0.01, 0.14]

The estimates *A* and *E* represent the proportion of the phenotypic correlation that is due to genetic (*A*) and environmental (*E*) variance, *r*_g_ = genetic correlations, *r*_e_ = environmental correlation

^*^p <0 .01

### Is there a direction of effect between family connectedness and self-control?

*Monozygotic twin within-individual change model*. The results show that, in genetically identical twin pairs, the twin showing the largest increase in family connectedness from age 14 to 16 did not report larger increase in self-control from age 14 to age 16 than the co-twin showing lower increase (or even decrease) in experienced family connectedness: *r* = 0.01, *p* = 0.83, 95% CI [− 0.08, 0.10].

## Discussion

The present study investigated the association between family connectedness and self-control, examining whether the association still holds in adolescence as well as the nature of this association. In line with the literature on early and middle childhood, the results confirmed that more reported family connectedness is related to better self-control in adolescents, albeit with a small effect size. When investigating the nature of this association, we found that this correlation was mainly explained by common genetic factors, with the effects of environmental factors being small. That is, the monozygotic twin who reports more family connectedness did not show higher self-control than his/her co-twin and, vice versa, the monozygotic twin with more self-control did not report more family connectedness than his/her co-twin. These findings are in line with results from middle childhood, where genetic factors play a role in explaining the overlap between family connectedness and child outcomes [12, 29, 35]. However, environmental factors play a larger role in early and middle childhood, while over time genetic factors increasingly play a role in spelling out individual differences in adolescent outcomes [42].

These findings suggest that while the association between family connectedness and self-control holds in adolescence, the two traits are not likely to causally influence one another over time. Rather, an underlying common factor such as genetic pleiotropy with some additional unique environmental influences seems to drive their association. Although there is common awareness that correlation does not equal causation, past research may have overestimated the association and too quickly concluded that the significant phenotypic relationship implies a transfer effect. For example, earlier studies investigating person–environment interactions emphasize how socializing processes are the driving source behind optimal self-control development [15, 34, 43]. The results of this study emphasize that understanding person–environment transactions are more complex, specifically highlighting the key role of biological factors in socializing processes. As such, if we aim to understand the mechanisms underlying person–environment transaction, it is important to incorporate both environmental and biological factors [42]. Combining these factors, thereby bridging multiple scientific disciplines, is necessary to paint a more complete picture of the etiology of individual differences in self-control.

An explanation for the role of shared genetic factors is that individuals evoke an environment as a result of their person characteristic: their self-control evokes more family connectedness and this is genetically mediated (evocative gene–environment correlation, [49]). Passive gene–environment correlation could also be a possible explanation, when there is a correlation between the environment the child is raised and the genotype a child inherits. For example, parents with high self-control are more likely to create a house environment with family connectedness but also transfer their ‘self-control’ genes to their children [49]. Perhaps gene–environment interaction is also at play, with certain genotypes being more sensitive to certain environments, positive or negative, as suggested by the diathesis stress model and the differential susceptibility theory [5, 44].

In this study, we quantified the extent to which environmental and genetic factors contribute to family connectedness, self-control and their overlap. While this approach is increasingly being applied in psychology, it is also of great use to other social sciences which traditionally do not include biological mechanisms (e.g., sociology, criminology, economics). Incorporating both genetic and environmental mechanisms is key, as without this understanding it is hard to pinpoint causal processes underlying phenomena we encounter [18]. Namely, the association between the social context (e.g., family connectedness) and child outcomes (e.g., self-control) can be the result of a true directional effect or, alternatively, be caused by common genetic factors simultaneously influencing both the family context and child outcomes (genetic pleiotropy or genetic confounding [48]). Not taking this alternative pathway into account potentially confounds research findings, hindering an attempt to reveal causal mechanisms explaining the outcome. While some researchers keep distrusting twin models (e.g., Burt and Simons [8]), years of cumulating evidence have shown the use of these models over and above the limitations these might have [50]. All traits are to a certain extent influenced by biological factors [51], and simply ignoring genetic influences would be naïve. As it would be naïve to only focus on genetic factors when investigating a child in its context. It is both nature and nurture contributing to individual differences in health and behavior, and incorporating both is an important way forward in our understanding of the way we become.

A limitation of the present study is that while we can investigate the nature of the association, we cannot unravel the more complex mechanisms underlying the association. As such, we do not know whether the association is explained by genetic pleiotropy, gene–environment correlation, gene–environment interaction, or a combination of these. Research designs such as children-of-twin designs, sibling designs, adoption studies, transmitted versus non-transmitted alleles, and interactions between polygenic risk scores and environments would allow for a deeper understanding of such mechanisms and are highly recommended for future research ([14, 29–39, 47, 65]). Another limitation of the study is that we focused a certain period in adolescence (i.e., adolescents aged 14 and 16). While the current approach allowed to examine cross-age effects of family connectedness on self-control and vice versa, longitudinal studies stretching across childhood would be an important next step in our understanding of the way adolescents develop within, and in interaction with, their social world. It is also important to emphasize that we only included self-reports, possibly inflating person-specific covariances due to single-informant non-random error, and we recommend replication of our findings in the future applying a multiple-rater approach.

Alternatively, the direction of causality (DoC, Duffy and Martin [20]; Heath et al. [32]) is a very elegant model of testing directionality. There are, however, a number of conditions to be met in order for this model to work. One main condition is that the correlation between the two traits (in this case family connectedness and self-control) should be moderate to strong. This is to ensure the model gives reliable prediction of the direction of causation. As the correlation between family connectedness and self-control was small, we do not have the confidence for this model to work well with the data. In the future, with more fine-grained molecular data at hand, methods such as Mendelian randomization and genomic SEM could be promising to further unravel the causal processes underlying self-control development [24, 40].

As a concluding note, it is important to emphasize that twin models assess variance differences which does not mean change [30]. So, while we do see that change occurs over time—family connectedness and self-control are significantly and positively associated over time—the current results demonstrate that most variance is explained by family members sharing the same genes. This implies that while we can still apply association studies such as correlational analyses and cross-lagged panel models, we should be more careful in the interpretation of the underlying causes of such associations. That is, phenomena we see within families can also be the product of parent and children sharing the same genes rather than being exclusively attributable to environmental processes.

## Electronic supplementary material

Below is the link to the electronic supplementary material.

Supplementary file1 (DOCX 16 kb)
