# Supplementary material for: Investigating the association between family connectedness and self-control in adolescence in a genetically sensitive design
Source: Eur Child Adolesc Psychiatry. 2020 Feb 5;29(12):1683–92. doi: 10.1007/s00787-020-01485-9 (PMC7641933; doi:10.1007/s00787-020-01485-9)
Supplement: Supplementary file 1 — Supplementary file1 (DOCX 16 kb) [file 787_2020_1485_MOESM1_ESM.docx]

| **Table S1**  Sample sizes for all effect sizes calculated in the manuscript split by zygosity | | | | | |
| --- | --- | --- | --- | --- | --- |
|  |  | **1.** | **2.** | **3.** | **4.** |
| 1. | Family Connectedness MZ twins age 14 | 3303 |  |  |  |
| 2. | Family Connectedness MZ twins age 16 | 1288 | 2377 |  |  |
| 3. | Self-control MZ twins age 14 | 3294 | 1323 | 3473 |  |
| 4. | Self-control MZ twins age 16 | 1418 | 2373 | 1459 | 2603 |
|  |  |  |  |  |  |
|  |  | **1.** | **2.** | **3.** | **4.** |
| 1. | Family Connectedness DZ twins age 14 | 5527 |  |  |  |
| 2. | Family Connectedness DZ twins age 16 | 1867 | 3620 |  |  |
| 3. | Self-control DZ twins age 14 | 5522 | 1931 | 5823 |  |
| 4. | Self-control DZ twins age 16 | 2045 | 3615 | 2115 | 3957 |

*Note*: sample sizes of the MZ twin were used in the MZ twin difference models. At age 14, 83% of the twin pairs were complete, at age 16 85% of the twin pairs were complete.

| **Table S2** Model fitting assessing means and variance differences for family connectedness and self-control age 14 and age 16, respectively | | | | | | | |
| --- | --- | --- | --- | --- | --- | --- | --- |
| **#** | **Model** | **EP** | **-2LL** | **Comp.** | **χ2** | **Δdf** | ***p* value** |
| **Age 14** | |  |  |  |  |  |  |
| 0 | Saturated | 70 | -43570.55 | - | - | - | - |
| 1 | Birth Order - Equal Means and Equal Variances | 54 | -43575.19 | 0 | 4.64 | 16 | 1.00 |
| 2 | Zygosity - Equal Means | 46 | -43582.87 | 1 | 7.68 | 8 | 0.96 |
| 3 | Zygosity - Equal Variances | 38 | -43587.38 | 2 | 4.51 | 8 | 0.81 |
| 4 | Gender - Equal Means | 36 | -43590.74 | 3 | 3.36 | 2 | 0.19 |
| 5 | Gender - Equal Variances | 34 | -43592.79 | 4 | 2.05 | 2 | 0.36 |
| 6 | MZm = Mzf & DZm = DZf | 22 | -43598.54 | 5 | 5.76 | 12 | 0.93 |
| 7 | DZ = Dos | 16 | -43601.91 | 6 | 3.36 | 6 | 0.76 |
|  |  |  |  |  |  |  |  |
| **Age 16** | |  |  |  |  |  |  |
| 0 | Saturated | 70 | -30150.85 | - | - | - | - |
| 1 | Birth Order - Equal Means and Equal Variances | 54 | -30157.68 | 0 | 6.83 | 16 | 0.98 |
| 2 | Zygosity - Equal Means | 46 | -30173.11 | 1 | 15.43 | 8 | 0.05 |
| 3 | Zygosity - Equal Variances | 38 | -30177.51 | 2 | 4.40 | 8 | 0.82 |
| 4 | Gender - Equal Means | 36 | -30182.00 | 3 | 4.49 | 2 | 0.81 |
| 5 | Gender - Equal Variances | 34 | -30186.61 | 4 | 4.61 | 2 | 0.10 |
| 6 | MZm = Mzf & DZm = DZf | 22 | -30196.62 | 5 | 10.01 | 12 | 0.62 |
| 7 | DZ = Dos | 16 | -30198.25 | 6 | 1.63 | 6 | 0.95 |
| *Note.* ep = estimated parameters, -2ll = minus 2 loglikelihood, Comp= model compared to, χ2 = chi square, df = degrees of freedom, MZm= monozygotic male twins, MZf= monozygotic female twins, DZm=dizygotic male twins, DZf=dizygotic female twins, Dos = dizygotic opposite gender twins. In model 6, the correlations between monozygotic males and monozygotic females, and the correlations between dizygotic males and females were constrained to be equal. In model 7, the correlations between dizygotic same-sex and opposite-sex were constrained to be equal. | | | | | | | |

|  |  |  |  |
| --- | --- | --- | --- |
| **Table S3** *Estimated parameters (EP) and model fit of bivariate twin models* | | |  |
| **Model** | **EP** | **RMSEA** | **CFI** |
| Bivariate twin model age 14 | 12 | 0.02 | 0.99 |
| Bivariate twin model age 16 | 12 | 0.02 | 0.98 |
| Bivariate twin model family connectedness age 14, self-control age 16 | 12 | 0.00 | 1.00 |
| Bivariate twin model self-control age 14, family connectedness age 16 | 12 | 0.00 | 1.00 |
| *Note*. RMSEA= Root Mean Square Error of Approximation (RMSEA), CFI= Comparative Fit Index | | | |
